# Diagnostic performance and interobserver agreement of O-RADS US v2022 and its combination with serum biomarkers in surgically treated epithelial ovarian-adnexal masses

**DOI:** 10.3389/fmed.2026.1930481

**Published:** 2026-09-11

**Authors:** Ying Li, Xiaoqin He, Liping Chen, Liping Zhong, Xiaohong Zhong, Yuning Lin

**Affiliations:** 1 Department of Ultrasound, Department of Obstetrics and Gynecology, Women and Children's Hospital, School of Medicine, Xiamen University, Xiamen, Fujian, China; 2 Medical Laboratory Center, Xiamen Humanity Hospital, Xiamen, Fujian, China

**Keywords:** CA125, diagnostic performance, HE4, O-RADS US v2022, ovarian-adnexal masses

## Abstract

**Objective:**

To evaluate the diagnostic performance of the American College of Radiology (ACR) Ovarian-Adnexal Reporting and Data System for Ultrasound (O-RADS US v2022) and a combined O-RADS US v2022–CA125–HE4 model in differentiating benign from borderline or malignant epithelial ovarian-adnexal masses, and to assess interobserver agreement for O-RADS US v2022 classification.

**Methods:**

This retrospective study included 282 histopathologically confirmed epithelial ovarian-adnexal lesions from 270 surgically treated patients between January 2022 and June 2025. Of the 282 lesions, 233 were benign and 49 were borderline/malignant, with borderline tumors included in the disease-positive group for the primary binary diagnostic analysis. Two sonographers with different levels of experience independently classified stored ultrasound images according to O-RADS US v2022. Categories 4-5 were prespecified as test-positive. Analyses of the combined O-RADS US v2022–CA125–HE4 model were restricted to 238 lesions with complete CA125 and HE4 data.

**Results:**

Among the 282 lesions, 233 (82.6%) were benign and 49 (17.4%) were borderline/malignant. In a sensitivity analysis excluding borderline tumors, all 20 invasive malignant lesions were classified as O-RADS US v2022 categories 4–5 by both sonographers. Using O-RADS US v2022 categories 4–5 as test-positive, Sonographer A achieved an AUC of 0.924 (95% CI, 0.892–0.955), with a sensitivity of 0.980, specificity of 0.670, PPV of 0.384, and NPV of 0.994. Sonographer B achieved an AUC of 0.904 (95% CI, 0.863–0.945), with a sensitivity of 0.980, specificity of 0.674, PPV of 0.387, and NPV of 0.994. The AUC was 0.769 (95% CI, 0.684-0.853) for CA125 alone and 0.689 (95% CI, 0.593–0.784) for HE4 alone. Among the 238 lesions with complete biomarker data, the combined O-RADS US v2022–CA125–HE4 model yielded an AUC of 0.937 (95% CI, 0.901–0.972), with a sensitivity of 0.721, specificity of 0.954, PPV of 0.775, and NPV of 0.939. Interobserver agreement for O-RADS US v2022 classification was excellent (linearly weighted κ = 0.963; 95% CI, 0.940–0.982).

**Conclusion:**

In this selected retrospective surgical cohort, O-RADS US v2022 demonstrated good diagnostic discrimination and excellent interobserver agreement. The combined O-RADS US v2022–CA125–HE4 model showed higher specificity but lower sensitivity than O-RADS US v2022 alone, without a statistically significant difference in AUC. Prospective external validation is required before clinical implementation.

## Introduction

Ovarian cancer (OC) remains one of the leading causes of cancer-related death among women with gynecological malignancies worldwide. According to GLOBOCAN 2022 estimates published by Bray et al. in 2024, 324,603 new ovarian cancer cases and 206,956 related deaths occurred worldwide in 2022 (1). In China, Sun et al. estimated approximately 61,100 new ovarian cancer cases and 32,600 related deaths in 2022, with age-standardized incidence and mortality rates of 5.68 and 2.64 per 100,000 population, respectively (2). Separately, a 2025 age-period-cohort analysis based on GBD 2021 data reported that the age-standardized incidence and mortality rates of ovarian cancer in China increased steadily after 2015 and were projected to continue rising through 2040 (3). Epithelial ovarian cancer (EOC) is the predominant histological type of malignant ovarian tumor, accounting for approximately 85%–90% of cases in general epidemiological studies. This distribution is also supported by a large Chinese retrospective cohort, in which epithelial tumors accounted for 871 of 1,016 malignant ovarian tumors (85.7%) (4, 5). In contrast, germ cell tumors and sex cord-stromal tumors are more common in children or young women, with distinct pathogenic mechanisms and clinical management pathways (6). Therefore, in the majority of adnexal mass cohort studies, malignant cases are predominantly of epithelial origin. Importantly, the current evidence supporting ultrasound-based risk stratification systems (e.g., IOTA, O-RADS) and serum biomarkers (CA125, HE4) is also primarily established in populations with epithelial tumors (7, 8). Consequently, this study focuses on epithelial ovarian-adnexal masses.

Transvaginal ultrasound (TVUS) is the first-line imaging modality for evaluating adnexal masses; however, conventional diagnosis largely relies on subjective experience, resulting in limited interobserver consistency. To improve the standardization of adnexal mass assessment, several structured risk-stratification tools have been developed, including the Risk of Malignancy Index, the IOTA Simple Rules, the ADNEX model, and the Ovarian-Adnexal Reporting and Data System (9, 10). Among these, O-RADS US provides a standardized lexicon, risk-stratification framework, and management recommendations for adnexal lesions. The present study focuses specifically on the diagnostic performance and interobserver agreement of O-RADS US v2022.

The original O-RADS US risk stratification and management system was published in the 2020 ACR consensus guideline (7). O-RADS US v2022 subsequently revised several descriptors and category assignments, including the introduction of bilocular cysts without solid components and acoustic shadowing in smooth solid lesions as features favoring lower risk, to improve risk-appropriate scoring (11). Subsequent studies have specifically evaluated O-RADS US v2022 and compared its diagnostic performance with earlier versions (12–14). Nevertheless, further validation in diverse populations remains necessary.

Meanwhile, serum biomarkers CA125 and HE4 are closely associated with epithelial ovarian cancer, but their diagnostic performance when used alone is limited. Recent studies suggest that combining imaging-based risk stratification with tumor biomarkers can enhance the differentiation of malignant adnexal lesions and improve re-stratification of intermediate-risk cases (13, 15–19). To date, fewer than ten published studies have specifically evaluated O-RADS US v2022 in Chinese populations, and only a limited number have investigated its combination with serum biomarkers (14, 20). Evidence regarding the simultaneous combination of O-RADS US v2022 with both CA125 and HE4 in epithelial ovarian-adnexal masses therefore remains limited (13, 21).

Therefore, this study aimed to evaluate the diagnostic performance and interobserver agreement of O-RADS US v2022 in epithelial ovarian-adnexal masses and to assess the incremental diagnostic value of adding serum CA125 and HE4 to O-RADS US v2022.

## Materials and methods

### Study design and ethical approval

This single-center retrospective study was conducted in strict accordance with the Declaration of Helsinki. The study protocol was approved by the Ethics Committee of the Women and Children's Hospital, Xiamen University (Approval No.: KY-2025-108-K01). Given the retrospective nature of the study and the strict de-identification of patient data, the Ethics Committee granted a waiver of informed consent. The clinical inclusion period extended from January 2022 to June 2025. The research data were accessed from December 1 to December 31, 2025, during which the literature review and manuscript preparation were also completed. Before data extraction, the eligibility criteria, variable definitions, and image-review procedures were predefined. The analytical cohort was retrospectively assembled from available institutional surgical, ultrasound, and histopathological records according to these criteria. No patient or lesion in the available eligible dataset was selectively included or excluded on the basis of anticipated diagnostic performance. The analytical dataset was locked before formal image review and statistical analysis, and both reviewers were blinded to the pathological findings during O-RADS assessment. This study was reported in accordance with the Standards for Reporting Diagnostic Accuracy Studies (STARD) 2015 guideline (22).

### Study population

Patients who underwent surgical treatment for an adnexal or pelvic mass at our hospital between January 2022 and June 2025 were retrospectively screened. Because epithelial histology cannot be determined with certainty before surgery, epithelial tumor status was not used as a preoperative screening criterion. Instead, postoperative histopathological findings were reviewed after surgery to determine eligibility for the final study cohort. Accordingly, the present study represents a retrospectively assembled surgical cohort restricted to target lesions histopathologically confirmed as epithelial ovarian-adnexal tumors. During the study period, 1,433 patients with postoperative histopathological records for an adnexal or pelvic mass were screened. Of these, 319 patients had target lesions histopathologically confirmed as epithelial ovarian-adnexal tumors. After application of the remaining eligibility criteria, 49 patients were excluded and 270 patients were included in the final study cohort.

Patients were eligible if they had: (1) undergone transvaginal and/or transabdominal gynecologic ultrasound at our institution within one month before surgery, with adequate images available in the PACS system; and (2) a definitive postoperative histopathological diagnosis of the target adnexal lesion.

Postoperative histopathological findings were first reviewed to identify patients with histopathologically confirmed epithelial ovarian-adnexal tumors. Patients whose target lesions were not histopathologically confirmed as epithelial ovarian-adnexal tumors were excluded at this stage. Among the 319 patients meeting the histopathological eligibility criterion, patients were excluded if they met any of the following remaining criteria: (1) pregnancy; (2) radiotherapy or chemotherapy before ultrasound examination or serum biomarker assessment; (3) documented renal insufficiency or renal failure in the discharge diagnosis; (4) no qualifying preoperative ultrasound examination within one month before surgery; or (5) incomplete or poor-quality ultrasound images that precluded reliable O-RADS US v2022 assessment. Coexisting endometriosis, chronic inflammatory changes, or other benign gynecologic conditions did not constitute exclusion criteria when the target lesion was histopathologically confirmed as an epithelial ovarian-adnexal tumor.

After application of these eligibility criteria, 270 patients with histopathologically confirmed epithelial ovarian-adnexal tumors were included. Among them, 258 patients had unilateral lesions and 12 had bilateral lesions, resulting in a total of 282 lesions included in the O-RADS US v2022 analysis. The primary analysis was performed at the lesion level because O-RADS US v2022 classification and histopathological diagnosis were assigned to individual lesions. For patients with bilateral disease, each lesion was evaluated independently; both lesions were included when both met the lesion-level eligibility criteria. Because CA125 and HE4 were measured at the patient level, the same preoperative biomarker values were assigned to both lesion records for patients with bilateral lesions. Analyses involving CA125, HE4, and the combined model were restricted to lesions with complete biomarker data. The participant flow is summarized in Supplementary Figure S1.

### Ultrasound examination and image analysis

Ultrasound Examination Protocol: Ultrasound examinations were performed using high-end diagnostic systems, including GE Voluson E10, GE Voluson E8, and Philips Q7. For patients with a history of sexual activity, routine transvaginal ultrasound (TVUS) was conducted. When the mass was large or located high in the pelvis, limiting transvaginal visualization, transabdominal ultrasound was used in combination. For patients without a history of sexual activity, transabdominal ultrasound was performed initially; if image quality was suboptimal or lesion visualization was inadequate, transrectal ultrasound was performed after obtaining informed consent from the patient (or guardian for minors). All ultrasound images were stored in the hospital's PACS workstation system. Cine loops were available for some examinations but were not systematically archived for all cases. When available, cine loops were reviewed as supplementary information; however, retrospective O-RADS US v2022 classification was based primarily on the stored static grayscale and color Doppler images.

### Ultrasound image quality control and inclusion criteria

Image Inclusion Standards: All examinations included in the study were required to meet predefined image-retention criteria. Grayscale images were required to clearly demonstrate the anatomical relationship of the lesion to the ovary and uterus, include at least two orthogonal planes, provide three-dimensional measurements or at minimum the maximum lesion diameter, and show key morphological features, including septations, solid components, and papillary projections when present. Images permitting assessment of ascites and peritoneal nodules, when present, were also required. Color Doppler images were required to permit assessment of vascularity within solid components or papillary projections when present. Because the ultrasound examinations were performed in routine clinical practice using three different ultrasound systems, Doppler parameters, including pulse-repetition frequency, wall filter, and gain, were not standardized to identical settings across all examinations. The O-RADS color score was assigned retrospectively and independently by each sonographer according to the degree of vascularity visible on the available stored color Doppler images. All images were required to have adequate resolution, appropriate scanning depth and visible body surface marker for reliable retrospective assessment.

Before formal O-RADS US v2022 classification, image adequacy was evaluated against the predefined image-retention criteria. Only examinations with sufficient stored grayscale and color Doppler images to assess the morphological and vascular descriptors required for retrospective O-RADS US v2022 classification were retained. Examinations with incomplete or poor-quality images that precluded reliable O-RADS US v2022 assessment were excluded before formal classification, and no O-RADS category was assigned. A total of 19 patients were excluded for this reason.

Image Interpretation Process: Two sonographers with different levels of experience—one senior (A, >10 years) and one junior (B, 4 years) in gynecologic ultrasound—independently reviewed the images retrospectively. Both sonographers completed the official ACR O-RADS online training course before the formal image review. No additional formal calibration exercise was performed before study assessment. Image review was performed independently. Both sonographers were blinded to histopathological findings, serum CA125 and HE4 values, clinical information, menopausal status, and the original ultrasound report; only the stored ultrasound images required for O-RADS US v2022 assessment were available during image review. For patients with multiple masses: Unilateral lesions: the mass with the most complex sonographic features in the affected adnexal region was selected for evaluation. Bilateral lesions: the most complex mass in each adnexal region was independently assessed. O-RADS US v2022 categories 4–5 were prespecified as test-positive for the primary binary diagnostic endpoint, whereas categories 1–3 were considered test-negative.

### Tumor marker assessment

Serum CA125 and HE4 levels measured within one week before surgery were extracted from the hospital electronic medical record database by two researchers and cross-checked to ensure data accuracy. CA125 and HE4 were measured using Roche e602/e801 analyzers (Roche Diagnostics) by electrochemiluminescence immunoassay (ECLIA). CA125 results were reported in U/mL and HE4 results in pmol/L. The institutional reference interval for CA125 was 0–35 U/mL, whereas the reference intervals for HE4 were <92.1 pmol/L for premenopausal women and <121 pmol/L for postmenopausal women.

All tumor marker measurements were performed according to the laboratory's standard quality-control procedures. Commercial Bio-Rad quality-control materials at two concentration levels (low and high) were analyzed together with patient samples. Assay performance was monitored using Levey-Jennings charts and Westgard multirule quality-control criteria. Analytical imprecision was monitored using the coefficient of variation (CV), with acceptable imprecision defined as CV <1/3 of the total allowable error (TEa). The laboratory also participated in external quality assessment/proficiency testing programs, with acceptable performance during the study period.

In the statistical analyses, CA125 and HE4 were treated as continuous variables in their original measurement units, and no biomarker-specific diagnostic cutoff was applied in the primary combined model. Although the institutional reference intervals for HE4 were menopause-specific, these reference limits were reported for clinical context only and were not used to dichotomize HE4 values or to define model positivity. Menopausal status was recorded as a baseline characteristic but was not incorporated into the primary combined model. Menopausal status, age, smoking status, coexisting endometriosis, and coexisting adnexal inflammatory disease were available from the electronic medical records. Because menopausal status may influence the interpretation of serum HE4 and CA125 concentrations, an additional sensitivity analysis was performed by adding menopausal status (premenopausal vs. postmenopausal) to the primary O-RADS US v2022-CA125-HE4 logistic regression model. The primary model was retained unchanged to avoid overparameterization. Smoking status was not entered into regression analysis because no variation in smoking status was observed in the study cohort.

Renal impairment was assessed from the discharge diagnoses recorded in the electronic medical record. Patients with a documented diagnosis of renal insufficiency or renal failure were excluded; no additional laboratory-based eGFR or serum creatinine threshold was retrospectively applied.

### Statistical analysis

Data were analyzed using SPSS version 26.0. Continuous variables were summarized as mean ± standard deviation or median (interquartile range), as appropriate, and categorical variables as number (percentage); missing data were reported explicitly. Postoperative histopathological diagnosis served as the reference standard. For the primary binary diagnostic analysis, histopathologically confirmed borderline tumors and invasive malignant tumors were considered disease-positive, whereas benign epithelial tumors were considered disease-negative. For ROC analysis, the full O-RADS US v2022 category was treated as an ordinal test variable, with observed categories ranging from 2 to 5 in the present cohort. The AUC was calculated using this ordinal score. For calculation of sensitivity, specificity, PPV, NPV, and accuracy, categories 4–5 were prespecified as test-positive, whereas categories 1–3 were considered test-negative; because no lesion was assigned to categories 0 or 1, this threshold effectively compared categories 4–5 with categories 2–3. Because borderline tumors are biologically and clinically distinct from invasive malignancies, additional subgroup analyses were performed separately for borderline tumors and invasive malignant tumors. The distribution of O-RADS US v2022 categories among borderline tumors was also summarized. A sensitivity analysis excluding all borderline tumors was performed to evaluate diagnostic performance for invasive malignant tumors vs. benign lesions. Sensitivity, specificity, PPV, NPV, and accuracy were calculated from the corresponding 2 × 2 contingency tables and are reported with exact 95% binomial confidence intervals (Clopper-Pearson method). Because this retrospective study included all eligible cases during the study period, no *a priori* sample-size calculation was performed; estimate precision was assessed using 95% confidence intervals. Interobserver agreement for O-RADS US v2022 classification between the two sonographers was assessed using Cohen's weighted *κ* with linear weighting because O-RADS categories are ordinal. The 95% confidence interval for weighted *κ* was estimated using 1,000 patient-level bootstrap resamples. Overall exact agreement and category-specific agreement were also calculated. For each O-RADS category, category-specific agreement was defined as the proportion of assignments to that category that were concordant between the two sonographers, relative to all assignments to that category made by either sonographer. Kappa values were interpreted as follows: 0.01–0.20, poor; 0.21–0.40, fair; 0.41–0.60, moderate; 0.61–0.80, good; and 0.81–1.00, excellent.

### Construction of the combined model

The combined O-RADS US v2022-CA125-HE4 model was constructed using multivariable binary logistic regression and was restricted to the 238 lesions with complete CA125 and HE4 measurements. Histopathological diagnosis was used as the dependent variable, with benign lesions coded as 0 and borderline/malignant lesions coded as 1. The O-RADS US v2022 score assigned by Sonographer A was entered as a single ordinal predictor using the assigned category value (observed range, 2–5), rather than being dichotomized or dummy-coded. Sonographer A, the senior sonographer with more than 10 years of gynecologic ultrasound experience, was used for construction of the primary combined model. To assess whether the incremental effect of biomarker integration depended on sonographer experience, an analogous secondary model was additionally constructed using the O-RADS US v2022 scores assigned by Sonographer B. Serum CA125 (U/mL) and HE4 (pmol/L) were entered as continuous variables in their original measurement units. As a sensitivity analysis addressing the potential influence of menopausal status on serum biomarker interpretation, the primary logistic regression model was refitted with menopausal status added as an additional binary covariate (premenopausal vs. postmenopausal). The adjusted model was compared with the primary model using a likelihood-ratio test, and the AUC was reported. This analysis was considered secondary and did not alter the prespecified primary model. The fitted logistic regression equation (with coefficients shown in rounded form) was: logit(*P*) = −13.2676 + 2.6714 × O-RADS + 0.010604 × CA125 + 0.014257 × HE4, where *P* represents the predicted probability of a borderline/malignant lesion. The predicted probability was calculated as *P* = exp[logit(*P*)]/{1 + exp[logit(*P*)]}. The optimal probability threshold was determined by maximizing the Youden index on the ROC curve. The unrounded optimal threshold was 0.300791 (reported as 0.301). Classification was performed using the unrounded threshold, with predicted probabilities ≥0.300791 classified as model-positive and probabilities <0.300791 classified as model-negative. To assess potential optimism in model discrimination, internal validation was performed using 1,000 patient-level bootstrap resamples. Patients rather than individual lesions were used as the resampling unit to preserve the within-patient correlation of bilateral lesions. In each bootstrap sample, the logistic regression model was refitted using the same predictors, and the AUC was calculated in both the bootstrap sample and the original dataset. The mean difference between these AUCs was used to estimate optimism, which was subtracted from the apparent AUC to obtain the optimism-corrected AUC.

### Comparison of ROC curves

To formally assess the incremental discriminatory value of adding CA125 and HE4 to O-RADS US v2022, paired ROC curves were compared using DeLong's test. Because the combined models required complete CA125 and HE4 measurements, all paired ROC comparisons were restricted to the same 238 complete-case lesions. For each sonographer, the AUC of the ordinal O-RADS US v2022 score alone was compared with the AUC of the predicted probability generated by the corresponding O-RADS US v2022-CA125-HE4 logistic regression model. Differences in AUC (ΔAUC) were calculated together with their 95% confidence intervals and two-sided *P* values. A *P* value <0.05 was considered statistically significant. Because 12 patients contributed bilateral lesions, a patient-level sensitivity analysis was additionally performed by retaining only one lesion per patient to assess the potential influence of within-patient correlation. For bilateral cases, the lesion with the higher O-RADS US v2022 category assigned by Sonographer A was retained; if both lesions had the same category, the larger lesion was selected.

### Reference standard

The reference standard was the histopathological diagnosis based on surgical specimens. Histopathological classification was performed according to the fifth edition of the WHO Classification of Female Genital Tumours (23). For the primary binary diagnostic analysis, borderline tumors and invasive malignant tumors were considered disease-positive, whereas benign epithelial tumors were considered disease-negative. This grouping was used solely to define the primary diagnostic endpoint and does not imply histopathological, biological, or clinical equivalence between borderline tumors and invasive carcinomas. Because borderline tumors differ from invasive malignancies in biological behavior and clinical management, subgroup analyses were performed separately for borderline and invasive malignant tumors, together with a sensitivity analysis excluding borderline tumors (24).

## Results

**Figure 1:**
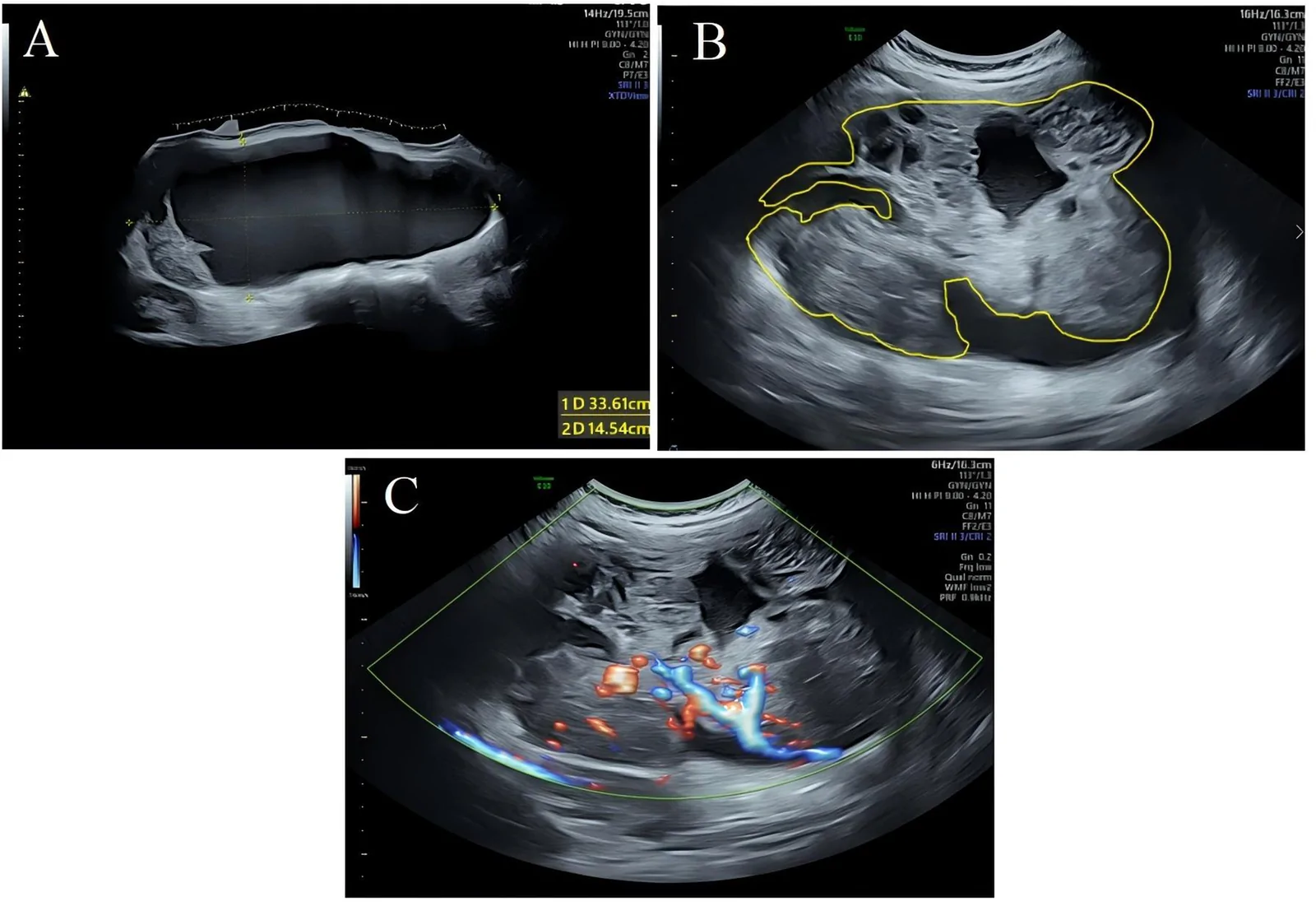
Representative ultrasound findings of a giant left ovarian mucinous borderline tumor. **(A)** Transabdominal extended-field-of-view image showing the maximum lesion diameter of 33.61 cm. **(B)** Grayscale ultrasound demonstrates a large complex cystic-solid mass. The extensive irregular solid component is outlined by the yellow line, with multiple small cystic spaces interspersed within the solid tissue. **(C)** Color Doppler image showing internal vascularity within the solid component. The lesion was classified as O-RADS US v2022 category 5 by both sonographers. Final postoperative histopathology confirmed a left ovarian mucinous borderline tumor.

**Figure 2:**
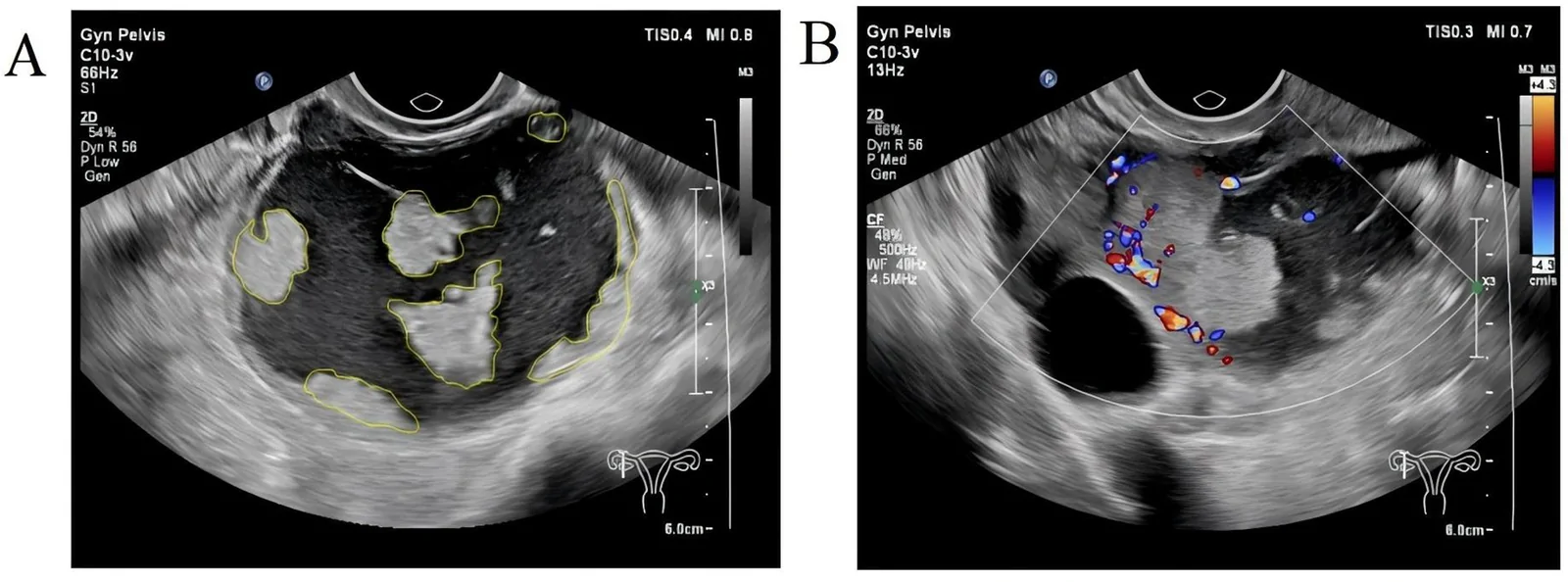
Serous borderline tumor of the right ovary, with dimensions of 6.9 cm × 4.5 cm × 5.8 cm. **(A)** Grayscale ultrasound demonstrates a complex cystic-solid mass. Multiple solid components are outlined by the yellow lines. **(B)** Color Doppler image showing internal vascularity within the solid component. Sonographer A classified it as O-RADS category 5, while Sonographer B also classified it as O-RADS category 5.

Patient age ranged from 10 to 73 years, with a median age of 36.0 years (IQR, 30.0–49.0 years). During the study period, 1,433 patients with postoperative histopathological records for an adnexal or pelvic mass were screened. Of these, 319 patients had target lesions histopathologically confirmed as epithelial ovarian-adnexal tumors. After exclusion of 49 patients who did not meet the remaining eligibility criteria, 270 patients were included in the final study cohort, contributing 282 epithelial ovarian-adnexal lesions. Participant selection is summarized in Supplementary Figure S1. Of the 282 lesions, 233 (82.6%) were benign and 49 (17.4%) were borderline or malignant, including 29 borderline tumors and 20 invasive malignant tumors. Baseline clinical and lesion characteristics according to histopathological outcome are summarized in Table 1, and detailed histopathological subtypes are presented in Table 2. At the patient level, 54 of 270 patients (20.0%) were postmenopausal. Postmenopausal status was present in 43 of 224 patients with only benign lesions (19.2%) and 11 of 46 patients with at least one borderline/malignant lesion (23.9%), with no significant difference between the two groups (Fisher's exact *P* = 0.543). Representative ultrasound images illustrating O-RADS US v2022 descriptors and corresponding histopathological diagnoses are shown in Figures 1, 2.

**Table 1:** Baseline clinical and lesion characteristics according to histopathological outcome.

Characteristic	Total (*n* = 282)	Benign (*n* = 233)	Borderline/malignant (*n* = 49)
Age, years, median (IQR)	36.0 (30.0–49.0)	36.0 (29.0–48.0)	36.0 (32.0–49.0)
Menopausal status, *n* (%)			
Premenopausal	225 (79.8%)	188 (80.7%)	37 (75.5%)
Postmenopausal	57 (20.2%)	45 (19.3%)	12 (24.5%)
Symptoms, *n* (%)			
No symptoms	184 (65.2%)	164 (70.4%)	20 (40.8%)
Lower abdominal pain	49 (17.4%)	37 (15.9%)	12 (24.5%)
Abdominal bloating	14 (5.0%)	7 (3.0%)	7 (14.3%)
Abnormal bleeding	12 (4.3%)	8 (3.4%)	4 (8.2%)
Palpable mass	7 (2.5%)	6 (2.6%)	1 (2.0%)
Other symptoms^a^	16 (5.7%)	11 (4.7%)	5 (10.2%)
Lesion size, cm, median (IQR)	7.3 (5.1–10.5)	7.3 (5.3–9.2)	9.6 (4.5–13.5)
Presentation, *n* (%)			
Unilateral	258 (91.5%)	216 (92.7%)	42 (85.7%)
Bilateral	24 (8.5%)	17 (7.3%)	7 (14.3%)
Ultrasound route, *n* (%)			
Transvaginal	199 (70.6%)	165 (70.8%)	34 (69.4%)
Transabdominal	33 (11.7%)	29 (12.4%)	4 (8.2%)
Transabdominal + transvaginal	45 (16.0%)	35 (15.0%)	10 (20.4%)
Transabdominal + transrectal	5 (1.8%)	4 (1.7%)	1 (2.0%)
CA125, U/mL, median (IQR)	16.5 (11.5–28.9)	14.6 (11.0–22.9)	43.4 (20.1–114.4)
Missing CA125, *n* (%)	33 (11.7%)	31 (13.3%)	2 (4.1%)
HE4, pmol/L, median (IQR)	46.8 (38.6–52.9)	45.8 (37.7–51.4)	51.8 (46.0–69.4)
Missing HE4, *n* (%)	41 (14.5%)	35 (15.0%)	6 (12.2%)
Histopathological category, *n* (%)			
Benign epithelial tumor	233 (82.6%)	233 (100%)	0
Borderline tumor	29 (10.3%)	0	29 (59.2%)
Invasive malignant tumor	20 (7.1%)	0	20 (40.8%)

Data are presented at the lesion level. IQR, interquartile range. CA125 and HE4 were available for 249 and 241 lesions, respectively.

a Other symptoms included irregular menstruation (*n* = 5), heavy menstrual bleeding (*n* = 5), urinary frequency (*n* = 3), lower abdominal distension (*n* = 2), and vaginal discharge (*n* = 1).

**Table 2:** Pathological types and proportions of epithelial ovarian-adnexal masses.

Tumor type	Histopathological subtype	Number (%)
Benign		233 (82.6%)
	Serous cystadenoma	99 (35.1%)
	Mucinous cystadenoma	92 (32.6%)
	Seromucinous cystadenoma	22 (7.8%)
	Serous cystadenofibroma	14 (5.0%)
	Benign Brenner tumor with mucinous cystadenoma	2 (0.7%)
	Mucinous cystadenofibroma	2 (0.7%)
	Brenner tumor	2 (0.7%)
Borderline tumors		29 (10.3%)
	Mucinous borderline tumor	13 (4.6%)
	Serous borderline tumor	8 (2.8%)
	Seromucinous borderline tumor	7 (2.5%)
	Endometrioid borderline tumor	1 (0.4%)
Invasive malignant tumors		20 (7.1%)
	Serous carcinoma	11 (3.9%)
	Endometrioid carcinoma	4 (1.4%)
	Clear cell carcinoma	3 (1.1%)
	Endometrioid carcinoma with undifferentiated carcinoma	1 (0.4%)
	Mucinous adenocarcinoma	1 (0.4%)
Total		282 (100.0%)

Percentages were calculated using the total number of lesions (*n* = 282) as the denominator.

### Interobserver agreement

Interobserver agreement for O-RADS US v2022 classification was assessed between Sonographer A (>10 years of gynecologic ultrasound experience) and Sonographer B (4 years of experience). Excellent interobserver agreement was observed between the two sonographers, with a linearly weighted *κ* of 0.963 (95% CI, 0.940–0.982). Exact agreement was achieved for 270 of 282 lesions (95.7%; 95% CI, 92.7%–97.8%), and all 12 discrepant ratings differed by only one O-RADS category. Among the 12 discrepant lesions, 7 were benign, 3 were borderline tumors, and 2 were invasive malignant tumors. Three disagreements crossed the prespecified category-4 diagnostic threshold, all of which involved benign lesions. Category-specific agreement was 97.2%, 94.2%, 96.0%, and 94.4% for O-RADS categories 2, 3, 4, and 5, respectively. The complete reader-by-reader classification matrix is presented in Table 3.

**Table 3:** Reader-by-reader cross-tabulation of O-RADS US v2022 categories.

Sonographer A	Sonographer B: 2	B: 3	B: 4	B: 5	Total
O-RADS 2	86	2	0	0	88
O-RADS 3	3	65	1	0	69
O-RADS 4	0	2	85	0	87
O-RADS 5	0	0	4	34	38
Total	89	69	90	34	282

Overall exact agreement was 270/282 (95.7%). Category-specific agreement represents the proportion of assignments to each O-RADS category that were concordant between the two sonographers. Category-specific agreement was 97.2%, 94.2%, 96.0%, and 94.4% for O-RADS categories 2, 3, 4, and 5, respectively. All discrepant ratings differed by only one O-RADS category.

### Diagnostic performance

**Figure 3:**
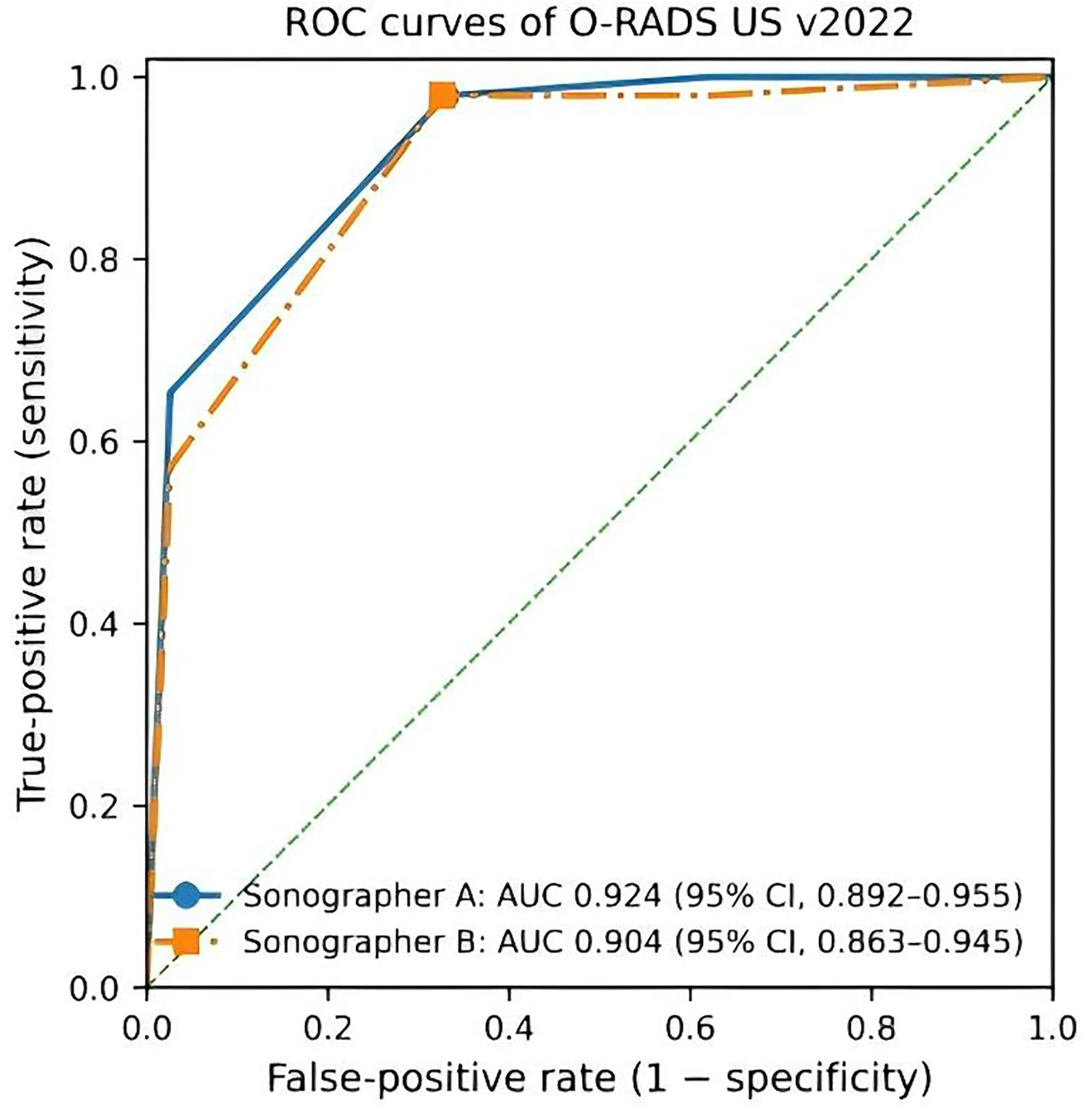
Receiver operating characteristic curves of O-RADS US v2022 for sonographers A and B. The analysis included 282 lesions, comprising 233 benign and 49 borderline/malignant lesions. Borderline and invasive malignant tumors were considered disease-positive. O-RADS US v2022 categories 2–5 were analyzed as an ordinal score; markers indicate the prespecified category 4 threshold (categories 4–5 test-positive).

No lesion was assigned to O-RADS US v2022 category 0 or 1 by either sonographer. Sonographer A assigned 88, 69, 87, and 38 lesions to categories 2, 3, 4, and 5, respectively, whereas Sonographer B assigned 89, 69, 90, and 34 lesions, respectively. The distribution of O-RADS categories according to histopathological outcome is shown in Table 4. Using the prespecified category 4 threshold (categories 4–5 as test-positive and categories 2–3 as test-negative), Sonographer A classified 48 of the 49 borderline/malignant lesions as positive and 156 of the 233 benign lesions as negative, yielding 48 true-positive (TP), 77 false-positive (FP), 156 true-negative (TN), and 1 false-negative (FN) results. The AUC for Sonographer A was 0.924 (95% CI, 0.892–0.955), with a sensitivity of 0.980 (95% CI, 0.891–0.999), specificity of 0.670 (95% CI, 0.605–0.730), PPV of 0.384 (95% CI, 0.298–0.475), NPV of 0.994 (95% CI, 0.965–1.000), and accuracy of 0.723 (95% CI, 0.667–0.775). Sonographer B yielded an AUC of 0.904 (95% CI, 0.863–0.945), with a sensitivity of 0.980 (95% CI, 0.891–0.999), specificity of 0.674 (95% CI, 0.610–0.734), PPV of 0.387 (95% CI, 0.301–0.479), NPV of 0.994 (95% CI, 0.965–1.000), and accuracy of 0.727 (95% CI, 0.671–0.778) (Figure 3).

**Table 4:** Distribution of histopathological outcomes according to O-RADS US v2022 category for each sonographer.

O-RADS category	Benign	Borderline	Invasive malignant	Total
Sonographer A				
2	88	0	0	88
3	68	1	0	69
4	71	13	3	87
5	6	15	17	38
Total	233	29	20	282
Sonographer B				
2	88	1	0	89
3	69	0	0	69
4	70	15	5	90
5	6	13	15	34
Total	233	29	20	282

**Figure 4:**
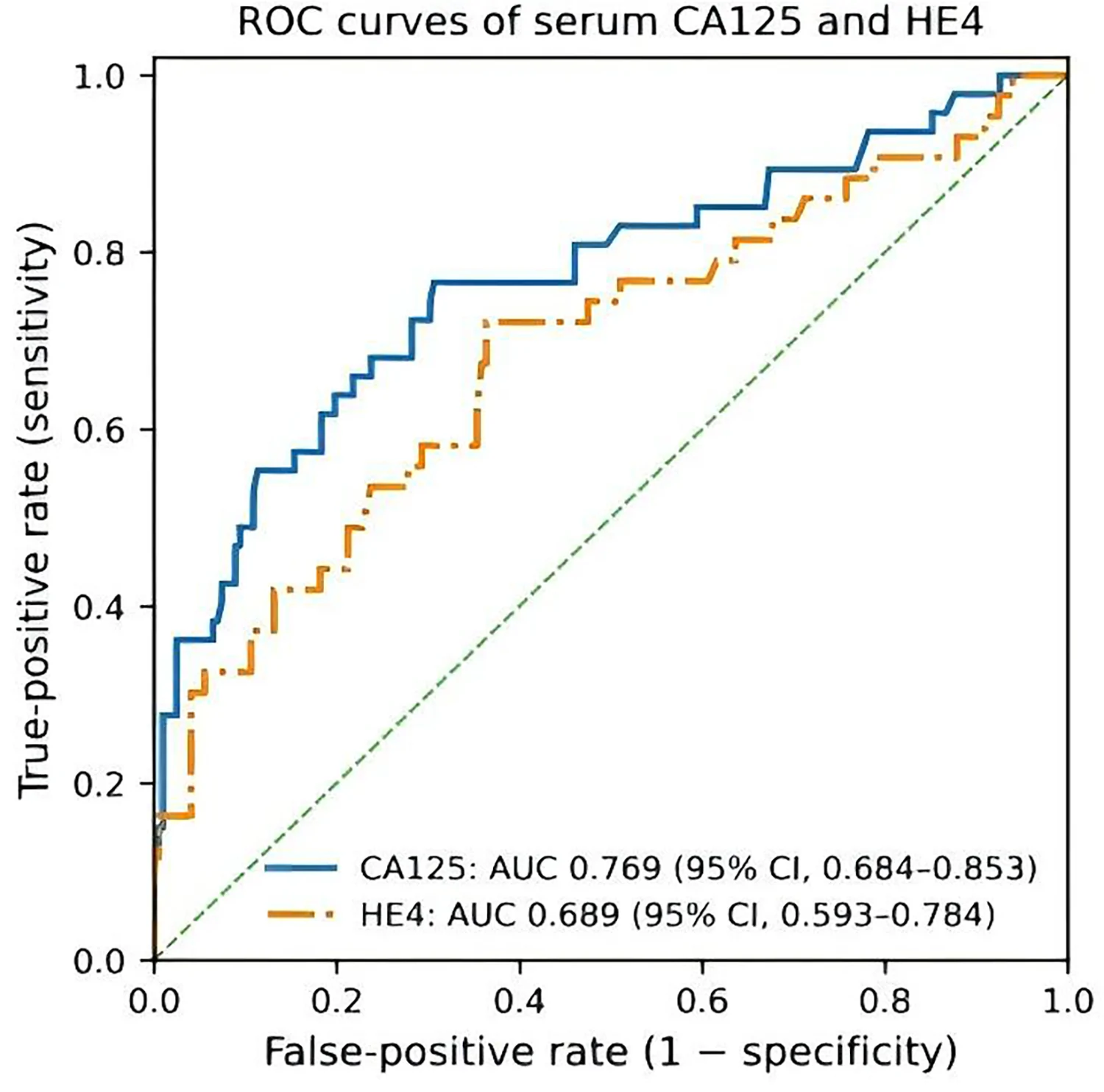
Receiver operating characteristic curves for serum CA125 and HE4. CA125 was available for 249 lesions (202 benign and 47 borderline/malignant), and HE4 was available for 241 lesions (198 benign and 43 borderline/malignant). Borderline and invasive malignant tumors were considered disease-positive. Both biomarkers were analyzed as continuous variables, with no prespecified biomarker-specific diagnostic cutoff.

**Figure 5:**
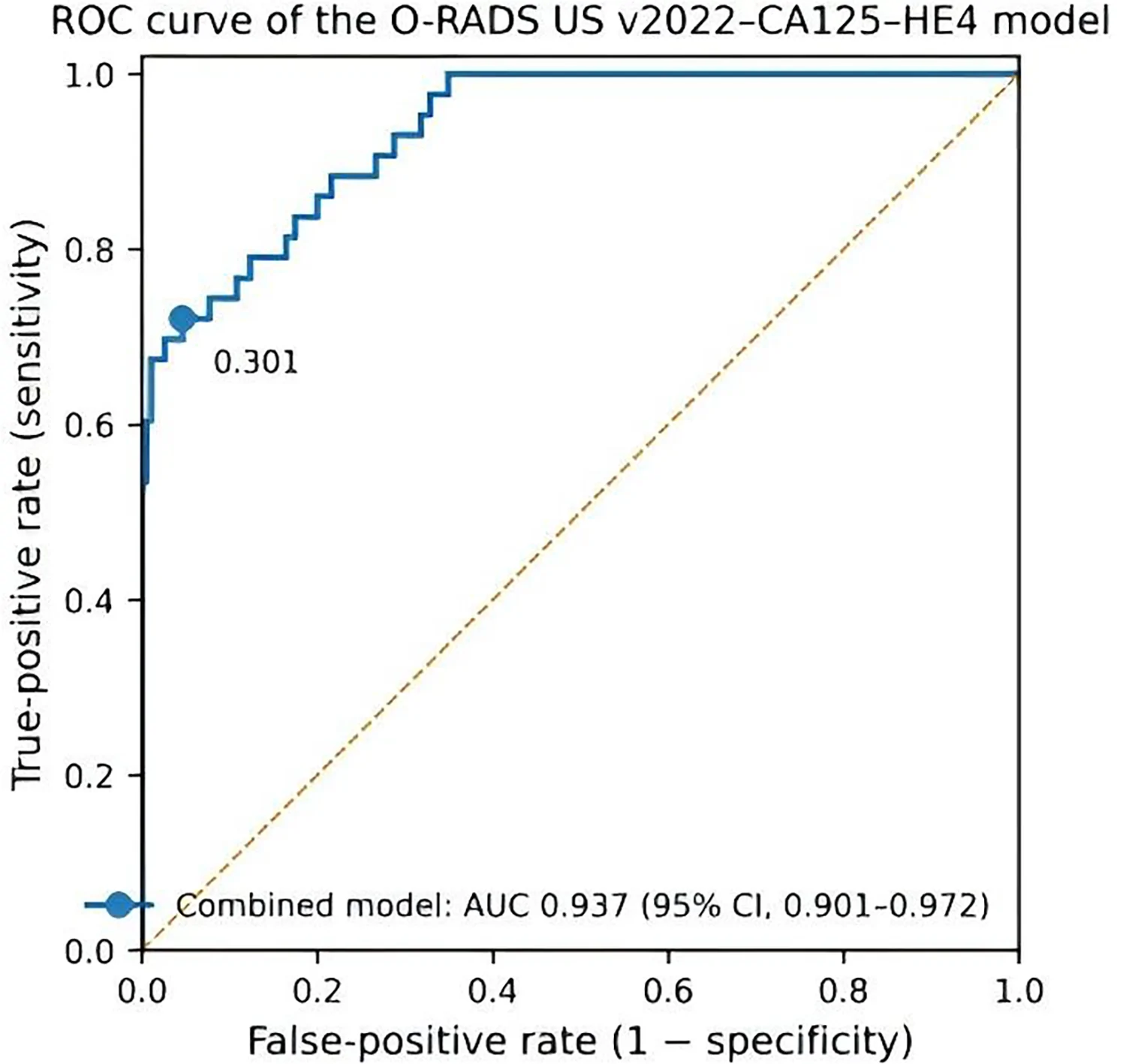
Receiver operating characteristic curve of the primary combined O-RADS US v2022-CA125-HE4 model. The analysis included 238 lesions with complete biomarker data, comprising 195 benign lesions and 43 borderline/malignant lesions. Borderline and invasive malignant tumors were considered disease-positive. The model included the Sonographer A O-RADS US v2022 category, CA125, and HE4. The AUC was 0.937 (95% CI, 0.901–0.972). The marked point indicates the Youden index threshold (predicted probability = 0.301).

Serum CA125 measurements were available for 249 lesions, including 202 benign and 47 borderline/malignant lesions. The AUC of CA125 alone was 0.769 (95% CI, 0.684–0.853). Serum HE4 measurements were available for 241 lesions, including 198 benign and 43 borderline/malignant lesions. The AUC of HE4 alone was 0.689 (95% CI, 0.593–0.784) (Figure 4). Complete CA125 and HE4 data were available for 238 lesions, comprising 195 benign and 43 borderline/malignant lesions. In this same complete-case cohort, threshold-based diagnostic performance of O-RADS US v2022 alone was recalculated using categories 4–5 as test-positive and categories 2–3 as test-negative. For Sonographer A, 42 lesions were true positive, 66 false positive, 129 true negative, and 1 false negative, yielding a sensitivity of 0.977 (95% CI, 0.877–0.999), specificity of 0.662 (95% CI, 0.590–0.728), PPV of 0.389 (95% CI, 0.297–0.487), NPV of 0.992 (95% CI, 0.958–1.000), accuracy of 0.718 (95% CI, 0.657–0.775), and an AUC of 0.926 (95% CI, 0.892–0.961). For Sonographer B, the corresponding counts were 42 true positive, 65 false positive, 130 true negative, and 1 false negative, yielding a sensitivity of 0.977 (95% CI, 0.877–0.999), specificity of 0.667 (95% CI, 0.596–0.732), PPV of 0.393 (95% CI, 0.300–0.492), NPV of 0.992 (95% CI, 0.958–1.000), accuracy of 0.723 (95% CI, 0.661–0.779), and an AUC of 0.912 (95% CI, 0.866–0.957) (Table 5). A multivariable binary logistic regression model was constructed using the O-RADS US v2022 score assigned by Sonographer A as an ordinal variable (observed range, 2–5) and serum CA125 and HE4 as continuous variables. The combined model yielded an AUC of 0.937. The Youden-optimal predicted-probability threshold was 0.300791 (reported as 0.301), and classification was based on the unrounded threshold. At this threshold, 31 lesions were classified as true positive, 9 as false positive, 186 as true negative, and 12 as false negative, corresponding to a sensitivity of 0.721 (95% CI, 0.563–0.847), specificity of 0.954 (95% CI, 0.914–0.979), PPV of 0.775 (95% CI, 0.615–0.892), NPV of 0.939 (95% CI, 0.897–0.968), and accuracy of 0.912 (95% CI, 0.868–0.945) (Figure 5). Among the 12 false-negative lesions, 10 were borderline tumors and 2 were invasive malignant tumors. The borderline false-negative lesions comprised five mucinous borderline tumors, three seromucinous borderline tumors, one serous borderline tumor, and one endometrioid borderline tumor. The two invasive malignant false-negative lesions comprised one serous carcinoma and one clear cell carcinoma. All 9 false-positive lesions were benign epithelial tumors, comprising five serous cystadenomas, three serous cystadenofibromas, and one seromucinous cystadenoma. The complete 2 × 2 classification results are summarized in Table 5, and the regression coefficients, odds ratios, and 95% confidence intervals are presented in Table 6. Internal validation using 1,000 patient-level bootstrap resamples showed only limited optimism in model discrimination; the apparent AUC was 0.937, the estimated optimism was 0.004, and the optimism-corrected AUC was 0.932. In a sensitivity analysis incorporating menopausal status into the primary O-RADS US v2022-CA125-HE4 model, the AUC was 0.942, compared with 0.937 for the primary model. Menopausal status was not independently associated with borderline/malignant histopathology after adjustment for O-RADS US v2022, CA125, and HE4 (OR, 0.319; 95% CI, 0.074–1.382; *P* = 0.127). Addition of menopausal status did not significantly improve model fit (likelihood-ratio *P* = 0.107), indicating that the principal findings of the combined model were robust to adjustment for menopausal status. To formally evaluate the incremental discriminatory value of adding CA125 and HE4, paired ROC comparisons were performed in the same 238 complete-case lesions. For Sonographer A, the AUC of O-RADS US v2022 alone was 0.926, compared with 0.937 for the corresponding combined model, yielding a ΔAUC of 0.010 (95% CI, −0.008 to 0.029; *P* = 0.283). For Sonographer B, the AUC of O-RADS US v2022 alone was 0.912, compared with 0.925 for the corresponding combined model, yielding a ΔAUC of 0.013 (95% CI, −0.008 to 0.034; *P* = 0.211). Thus, although the combined models showed numerically higher AUCs, neither comparison demonstrated a statistically significant incremental improvement in discrimination after addition of CA125 and HE4 (Table 7). A patient-level sensitivity analysis retaining only one lesion per patient included 270 independent patients. The AUCs were 0.921 for Sonographer A and 0.900 for Sonographer B, compared with 0.924 and 0.904, respectively, in the primary lesion-level analysis. Among the 226 patients with complete CA125 and HE4 data, the primary combined model yielded an AUC of 0.933, with a sensitivity of 0.725, specificity of 0.952, PPV of 0.763, NPV of 0.941, and accuracy of 0.912. These estimates were closely comparable to the primary lesion-level findings, suggesting that the inclusion of bilateral lesions had only a limited influence on the main results.

**Table 5:** Diagnostic performance of O-RADS US v2022 in the full and complete-case cohorts and of the combined model.

Diagnostic approach	*n*	TP	FP	TN	FN	Sensitivity (95% CI)	Specificity (95% CI)	PPV (95% CI)	NPV (95% CI)	Accuracy (95% CI)	AUC (95% CI)
O-RADS US v2022, Sonographer A, full cohort	282	48	77	156	1	0.980 (0.891–0.999)	0.670 (0.605–0.730)	0.384 (0.298–0.475)	0.994 (0.965–1.000)	0.723 (0.667–0.775)	0.924 (0.892–0.955)
O-RADS US v2022, Sonographer B, full cohort	282	48	76	157	1	0.980 (0.891–0.999)	0.674 (0.610–0.734)	0.387 (0.301–0.479)	0.994 (0.965–1.000)	0.727 (0.671–0.778)	0.904 (0.863–0.945)
O-RADS US v2022, Sonographer A, complete-case cohort	238	42	66	129	1	0.977 (0.877–0.999)	0.662 (0.590–0.728)	0.389 (0.297–0.487)	0.992 (0.958–1.000)	0.718 (0.657–0.775)	0.926 (0.892–0.961)
O-RADS US v2022, Sonographer B, complete-case cohort	238	42	65	130	1	0.977 (0.877–0.999)	0.667 (0.596–0.732)	0.393 (0.300–0.492)	0.992 (0.958–1.000)	0.723 (0.661–0.779)	0.912 (0.866–0.957)
O-RADS US v2022 (Sonographer A) + CA125 + HE4	238	31	9	186	12	0.721 (0.563–0.847)	0.954 (0.914–0.979)	0.775 (0.615–0.892)	0.939 (0.897–0.968)	0.912 (0.868–0.945)	0.937 (0.901–0.972)

Borderline and invasive malignant tumors were considered disease-positive. O-RADS US v2022 categories 4–5 were test-positive and categories 2–3 test-negative. The combined model used the unrounded Youden-optimal predicted-probability threshold of 0.300791 (reported as 0.301). CI, confidence interval; PPV, positive predictive value; NPV, negative predictive value; AUC, area under the curve.

**Table 6:** Multivariable logistic regression model combining sonographer A O-RADS US v2022, CA125, and HE4.

Variable	*β* coefficient	SE	Odds ratio	95% CI for OR	*P* value
Intercept	−13.268	2.044	–	–	<0.001
O-RADS US v2022 score	2.671	0.461	14.46	5.863–35.664	<0.001
CA125, U/mL	0.0106	0.0066	1.011	0.998–1.024	0.106
HE4, pmol/L	0.0143	0.0163	1.014	0.983–1.047	0.381

The dependent variable was histopathological diagnosis (0 = benign; 1 = borderline/malignant). O-RADS US v2022 was entered as a single ordinal variable using the assigned category value (observed range, 2–5); CA125 and HE4 were entered as continuous variables in their original units. OR, odds ratio; CI, confidence interval; SE, standard error.

**Table 7:** Paired comparison of ROC curves for O-RADS US v2022 alone and the combined O-RADS US v2022-CA125-HE4 models in the complete-case cohort.

Sonographer	*n*	O-RADS alone AUC	Combined-model AUC	ΔAUC	95% CI for ΔAUC	*P* value
Sonographer A	238	0.926	0.937	0.010	−0.008 to 0.029	0.283
Sonographer B	238	0.912	0.925	0.013	−0.008 to 0.034	0.211

Paired DeLong comparisons were restricted to the same 238 lesions with complete CA125 and HE4 measurements. ΔAUC represents the AUC of the combined model minus that of O-RADS US v2022 alone. CI, confidence interval; AUC, area under the curve.

When borderline and invasive malignant tumors were considered separately, 28 of 29 borderline tumors (96.6%; 95% CI, 82.2%–99.9%) were classified as O-RADS US v2022 categories 4–5 by each sonographer. Sonographer A assigned 1, 13, and 15 borderline tumors to categories 3, 4, and 5, respectively, whereas Sonographer B assigned 1, 15, and 13 borderline tumors to categories 2, 4, and 5, respectively. In contrast, all 20 invasive malignant tumors were classified as categories 4–5 by both sonographers. The AUCs for borderline tumors vs. benign lesions were 0.897 (95% CI, 0.853–0.942) and 0.875 (95% CI, 0.815–0.935) for Sonographers A and B, respectively. In the sensitivity analysis excluding all borderline tumors, the AUCs for distinguishing the 20 invasive malignant tumors from the 233 benign lesions were 0.962 (95% CI, 0.933–0.991) and 0.946 (95% CI, 0.912–0.981), respectively, with a sensitivity of 1.000 (95% CI, 0.832–1.000) for both sonographers.

## Discussion

Transvaginal ultrasound is the first-line imaging modality for evaluating adnexal masses. However, ultrasound assessment may be influenced by operator experience, and variability in terminology and risk communication can affect clinical interpretation and management. Moreover, there are issues such as inconsistent terminology in ultrasound reports and unclear risk expressions, which affect clinical triage and management decisions. One of the reasons for the proposal of O-RADS US v2022 is to solve the long-standing problems of “inconsistent terminology, poor report comparability, and result dependence on experience” in the ultrasound assessment of ovarian-adnexal masses. The ACR O-RADS committee has established a standardized dictionary and a structured risk stratification framework to define imaging features, risk classification, and corresponding management recommendations, thereby improving report consistency and interdisciplinary communication efficiency (25). The subsequently released O-RADS US risk stratification system and the v2022 update version have further optimized the classification logic for low-risk signs, aiming to improve specificity while maintaining high sensitivity, and enhance the stability of the system among operators with different levels of experience (7, 11). Previous studies have confirmed that O-RADS has excellent inter-operator consistency. Multiple external validation studies have reported that its classification consistency has reached the level of “substantial consistency” or even “excellent consistency” (26–28). In this study, all included cases met predefined image-retention and quality criteria, and two gynecologic sonographers with different levels of experience independently reviewed the stored images after completing the official ACR O-RADS online training course. Excellent interobserver agreement was observed (linearly weighted *κ* = 0.963; 95% CI, 0.940–0.982), suggesting that the structured lexicon and classification framework of O-RADS US v2022 can support reproducible risk stratification across readers with different levels of experience. Nevertheless, the magnitude of agreement should be interpreted in the context of the study design. Both sonographers had received formal O-RADS training and retrospectively evaluated stored images that fulfilled predefined quality criteria. Therefore, the observed agreement may be higher than that achievable during routine real-time ultrasound examinations, in which lesion morphology, mobility, vascularity, and other dynamic features are assessed during image acquisition. In addition, because this study was conducted at a tertiary referral center, the reproducibility and diagnostic performance of O-RADS US v2022 in primary-care or community settings were not directly evaluated and require further prospective validation.

Early validation studies of O-RADS have generally emphasized the importance of maintaining high sensitivity to minimize missed malignant lesions, although this may be accompanied by reduced specificity and an increased number of false-positive classifications (12). Liu et al. reported that O-RADS US v2022 maintained high diagnostic performance compared with the previous version and demonstrated excellent interobserver agreement (13). In the present study, using O-RADS US v2022 categories 4–5 as the test-positive threshold, both sonographers achieved a sensitivity of 0.980 and an NPV of 0.994, whereas specificity was 0.670 for Sonographer A and 0.674 for Sonographer B, with corresponding PPVs of 0.384 and 0.387, respectively. Only one of the 49 borderline/malignant lesions was classified as test-negative by each reader; however, 77 and 76 benign lesions were classified as test-positive by Sonographers A and B, respectively. These findings indicate that, at the prespecified category 4–5 threshold, the principal diagnostic strength of O-RADS US v2022 in our cohort was its very high sensitivity and NPV rather than high specificity. The relatively low PPV indicates that a substantial proportion of O-RADS-positive lesions were ultimately benign on histopathology. Some benign epithelial tumors may exhibit complex multilocular architecture, solid-appearing components, papillary projections, or increased vascularity, thereby meeting O-RADS descriptors associated with higher-risk categories despite benign histopathology. These cases illustrate the intended sensitivity-specificity trade-off of the category 4–5 threshold and emphasize that O-RADS risk stratification should not be interpreted as a pathological diagnosis. Because PPV and NPV are influenced by disease prevalence, the very high NPV and relatively low PPV should be interpreted in the context of the 17.4% prevalence of borderline/malignant lesions in this surgically treated cohort. Nevertheless, the AUCs of 0.924 for Sonographer A and 0.904 for Sonographer B indicate good overall discrimination. Taken together, these findings support O-RADS US v2022 as a standardized risk-stratification system with strong sensitivity for identifying borderline/malignant epithelial ovarian-adnexal lesions, while emphasizing that a positive O-RADS classification should not be regarded as equivalent to a pathological diagnosis of malignancy (29–31). Borderline tumors should nevertheless be considered separately from invasive malignancies because of their distinct biological behavior and clinical management. In the present study, borderline and invasive malignant tumors were combined only for the primary binary diagnostic endpoint and were not considered clinically equivalent. Subgroup analysis showed that 28 of 29 borderline tumors were classified as O-RADS US v2022 categories 4–5 by each reader, whereas all 20 invasive malignant tumors were classified as categories 4–5. After exclusion of borderline tumors, both sonographers achieved a sensitivity of 1.000 for invasive malignancy, with AUCs of 0.962 and 0.946 for Sonographers A and B, respectively. These findings indicate that borderline tumors represented a more challenging subgroup and support separate interpretation of borderline and invasive malignant lesions.

Serum CA125 and HE4 provide biological information that is complementary to the morphological risk assessment provided by O-RADS US v2022 (16, 21, 32, 33). In the present study, CA125 alone yielded an AUC of 0.769, whereas HE4 alone yielded an AUC of 0.689, indicating that neither biomarker alone achieved the discriminatory performance observed with O-RADS US v2022. Complete CA125 and HE4 measurements were available for 238 lesions, and the combined O-RADS US v2022-CA125-HE4 model was therefore evaluated in this complete-case cohort. The primary combined model based on Sonographer A yielded an AUC of 0.937.

Importantly, formal paired ROC comparisons did not demonstrate a statistically significant incremental improvement in discrimination after addition of CA125 and HE4. In the same 238 complete-case lesions, the AUC increased from 0.926 to 0.937 for Sonographer A (ΔAUC = 0.010, 95% CI, −0.008 to 0.029; *P* = 0.283) and from 0.912 to 0.925 for Sonographer B (ΔAUC = 0.013, 95% CI, −0.008 to 0.034; *P* = 0.211). Thus, although the combined models showed numerically higher AUCs for both sonographers, the present data do not demonstrate a statistically significant improvement in overall discriminatory performance attributable to the addition of CA125 and HE4.

Nevertheless, the combined model demonstrated a different sensitivity-specificity trade-off compared with O-RADS US v2022 alone. Because the probability threshold for the combined model was selected by maximizing the Youden index in the development cohort, whereas O-RADS US v2022 was evaluated using the prespecified category 4–5 threshold, the higher specificity and PPV observed for the combined model should not be interpreted as being directly attributable to biomarker integration. A more stringent O-RADS threshold could potentially produce a similar sensitivity-specificity trade-off. Importantly, paired ROC comparisons demonstrated no statistically significant incremental improvement in AUC after addition of CA125 and HE4. Therefore, CA125 and HE4 may provide complementary information for risk refinement, but the present findings do not demonstrate statistically significant incremental diagnostic value, and the combined model should not be regarded as a replacement for the high-sensitivity O-RADS US v2022 assessment.

Internal validation using 1,000 patient-level bootstrap resamples indicated only limited optimism in model discrimination, with an optimism-corrected AUC of 0.932 compared with the apparent AUC of 0.937. Nevertheless, internal validation does not replace independent external validation, particularly given the single-center retrospective design of the present study. Prospective external validation is required before this combined approach can be recommended for routine clinical decision-making.

This study has several limitations. First, the retrospective, single-center design and restriction to surgically treated, histopathologically confirmed epithelial lesions introduced substantial selection and spectrum bias. Common benign adnexal lesions outside the target epithelial-tumor spectrum, such as endometriomas, mature teratomas, fibromas, and functional cysts, were not represented in the final cohort. This restricted disease spectrum may have influenced the observed diagnostic performance compared with that expected in an unselected clinical population, in which non-epithelial benign lesions, functional cysts, and conservatively managed adnexal masses are commonly encountered. Therefore, the diagnostic performance observed in this study should not be extrapolated to the broader spectrum of adnexal masses encountered in routine ultrasound practice, including conservatively managed lesions. The relatively small number of disease-positive lesions, particularly the 20 invasive malignancies, also limits the precision of subgroup estimates. Second, O-RADS US v2022 assessment was based on retrospective review of stored images rather than on real-time ultrasound examinations. Cine loops were not systematically available for all examinations, and Doppler acquisition parameters were not standardized to identical settings across the three ultrasound systems. The O-RADS color score was therefore assigned retrospectively from the available stored color Doppler images. Dynamic features requiring real-time assessment, such as lesion mobility, could not always be independently reassessed from the archived examinations. Furthermore, 19 patients with incomplete or poor-quality ultrasound documentation were excluded before formal O-RADS classification. This requirement may have preferentially retained well-documented examinations and introduced information and selection bias, potentially overestimating both diagnostic performance and interobserver agreement. In addition, both sonographers had completed formal O-RADS training before image review, which may also have contributed to the high observed agreement. Third, the primary analyses were performed at the lesion level, and 12 patients contributed bilateral lesions, resulting in potential within-patient correlation. However, the patient-level sensitivity analysis produced results closely comparable to the primary analysis, suggesting that the influence of this clustering on the main findings was limited. Fourth, analyses of the combined O-RADS US v2022-CA125-HE4 model were restricted to the 238 lesions with complete biomarker data, and complete-case analysis may have introduced bias if biomarker missingness was not completely random. Finally, the combined model was developed and evaluated within the same retrospective cohort without independent external validation; therefore, its apparent diagnostic performance may be optimistic. Further prospective, multicenter studies using standardized real-time image acquisition and independent validation cohorts are warranted to confirm the reproducibility and clinical utility of these findings.

## Conclusion

In this selected retrospective surgical cohort of histopathologically confirmed epithelial ovarian-adnexal masses, O-RADS US v2022 demonstrated good overall diagnostic discrimination and excellent interobserver agreement. At the prespecified category 4–5 threshold, O-RADS US v2022 showed very high sensitivity and NPV but moderate specificity and PPV. The combined O-RADS US v2022-CA125-HE4 model showed a different sensitivity-specificity trade-off at its Youden-optimized probability threshold; however, because the two approaches were evaluated using different threshold-selection strategies, the higher specificity and PPV observed for the combined model cannot be attributed directly to biomarker integration. Importantly, addition of CA125 and HE4 did not result in a statistically significant increase in AUC. Subgroup analysis further showed that all invasive malignant lesions were identified by O-RADS US v2022 at the category 4–5 threshold, whereas one borderline tumor was classified below this threshold. CA125 and HE4 may provide complementary information for risk refinement, but no statistically significant incremental diagnostic value was demonstrated, and prospective multicenter external validation is required before routine clinical application.

## Data Availability

The original contributions presented in the study are included in the article/Supplementary Material, further inquiries can be directed to the corresponding authors.
